# Lubricity of gold nanocrystals on graphene measured using quartz crystal microbalance

**DOI:** 10.1038/srep31837

**Published:** 2016-08-24

**Authors:** M. S. Lodge, C. Tang, B. T. Blue, W. A. Hubbard, A. Martini, B. D. Dawson, M. Ishigami

**Affiliations:** 1 Department of Physics and NanoScience Technology Center, University of Central Florida, Orlando, FL 32816, USA; 2 School of Engineering, University of California Merced, Merced, CA 95343, USA; 3 Department of Physics and California NanoSystems Institute, University of California, Los Angeles, California 90095, USA

## Abstract

In order to test recently predicted ballistic nanofriction (ultra-low drag and enhanced lubricity) of gold nanocrystals on graphite at high surface speeds, we use the quartz microbalance technique to measure the impact of deposition of gold nanocrystals on graphene. We analyze our measurements of changes in frequency and dissipation induced by nanocrystals using a framework developed for friction of adatoms on various surfaces. We find the lubricity of gold nanocrystals on graphene to be even higher than that predicted for the ballistic nanofriction, confirming the enhanced lubricity predicted at high surface speeds. Our complementary molecular dynamics simulations indicate that such high lubricity is due to the interaction strength between gold nanocrystals and graphene being lower than previously assumed for gold nanocrystals and graphite.

A simple phenomenological formula, F = μN, where μ is the coefficient of friction and N is the normal force, governs macroscopic friction. Nanoscale contacts between surface asperities are assumed to determine μ, but the fundamental science of friction at nanoscale, nanotribology, is still in its infancy and many unsolved problems remain.

One of the most promising discoveries in nanotribology is structural lubricity^1^: low friction between atomically incommensurate surfaces. Structural lubricity was first anticipated in theoretical considerations of weakly-coupled incommensurate structures^2^,^3^ and has been observed on various surfaces such as mica^4^ as well as graphite and graphene^5^,^6^,^7^,^8^,^9^. Graphene offers a unique opportunity to extend such structural lubricity to the macroscale because it can be produced at television-screen dimensions^10^ and transferred onto arbitrary surfaces^11^,^12^,^13^ to reduce friction on a wide array of mechanical components. Indeed, recent experiments have already shown that graphene can be used to reduce friction of macroscopic steel-to-steel contacts^14^,^15^,^16^ as well as diamond-like carbon on SiO_2_^17^.

Recent calculations have shown that gold nanocrystals on graphite possess unusually low friction^18^,^19^. Two friction regimes are predicted to exist at different speeds. At low speeds, nanocrystals on graphite are expected to possess higher friction, consistent with previous studies of thermal diffusion of gold on graphite^20^ and on graphene^21^. However, at high sliding speeds in the range of 100 m/sec, nanocrystals are expected to behave radically differently, with much smaller drag and, therefore, lower friction. Such high speeds are not easily accessible by atomic force microscopy (AFM)^22^,^23^, and the use of a quartz crystal microbalance (QCM) was proposed to confirm this enhanced lubricity predicted at high surface speeds^18^,^19^. QCMs oscillate in shear mode when driven at their resonant frequency. Their surface velocity is given by 2π*fA*sin(2π*ft*), where *A*_*osc*_ is the amplitude, *f* is the resonant frequency and *t* is the elapsed time. The oscillation amplitude is given by *A*_*osc*_ = *C* × *Q* × *V*, where *C* = 1.3 pm/V, *Q* is the quality factor, and V is the driving oscillation voltage^24^. Peak velocities can reach m/sec with displacement amplitudes in the range of 1–10 nm. The high quality factors of QCMs make them extremely sensitive to mass adsorption and to changes in dissipation at their surfaces. In the formalism developed for analyzing viscous friction of adsorbates on QCMs^25^,^26^, the change in frequency and dissipation induced by adsorbates can be connected to the slip time, *τ*_*s*_, which is the characteristic time required for sliding adsorbates to stop, by the formula


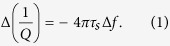


The slip time is related to the drag coefficient as *τ*_*s*_ = *ρ*/*ξ*, where *ξ* is the drag coefficient and *ρ* is the mass per unit area of adsorbates. QCMs along with this formalism have been exploited to determine the frictional behavior of weakly adsorbed monatomic gases like Xe and Kr^25^,^26^,^27^, as well as the friction and adhesion of micron-sized beads^28^,^29^. The technique has also recently been used to measure friction of adsorbed gases on graphene^30^,^31^,^32^ as well as on graphite^33^. However, QCMs have not yet been used to measure the friction of nanometer-scale, crystalline adsorbates.

We observe that depositing gold nanocrystals on graphene-coated QCMs decreases the resonance frequency and increases the energy dissipation. The measured changes of frequency and dissipation induced by gold nanocrystals are used to determine the slip time of the nanocrystals on graphene using the framework previously developed for friction of adatoms^25^,^26^. The determined slip time is found to be more than an order of magnitude higher than the value predicted for ballistic friction, confirming the enhanced lubricity predicted at high surface speeds. Our molecular dynamics calculations indicate that the observed lubricity exceeds the previous predictions because the interaction strength between gold nanocrystals and graphene is lower than the value used for those predictions.

## Results

**Figure 1:**
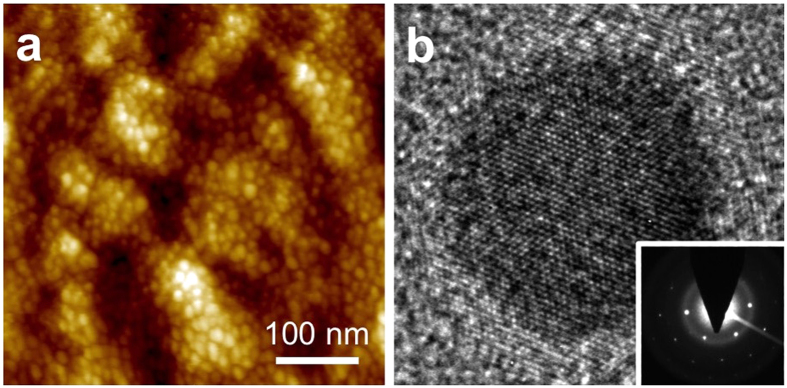
(**a**) AFM image of gold nanocrystals on the QCM with graphene. (**b**) High resolution transmission electron microscopy image of a representative single gold nanocrystal on graphene. (Inset) Diffraction pattern of the nanocrystal showing the fcc crystal structure.

Gold is deposited using electron beam evaporation after stabilizing a QCM coated with graphene and a control QCM without graphene in an ultra-high vacuum (UHV) environment. The areal density of nanocrystals is determined using an AFM after QCM measurements to be 1.01 ± 0.02 × 10^12^ crystals/cm^2^ as shown in Fig. 1a. This is nearly two orders of magnitude higher than the defect densities in graphene as determined from Raman spectroscopy, which was 1.9 × 10^10^ defects/cm^2^. As such, we expect the responses of the graphene QCM to be dominated by the interaction of nanocrystals with the graphene lattice. In AFM images, nanocrystals appear to be disk-shaped with large apparent lateral diameter and smaller height with the height ranging from 2.8 to 4.0 nm. The apparent shape is due to the convolution between the nanocrystals and the AFM tip shape. As shown in Fig. 1b, nanocrystals are found to be isotropic in shape from transmission electron microscopy (TEM).

**Figure 2:**
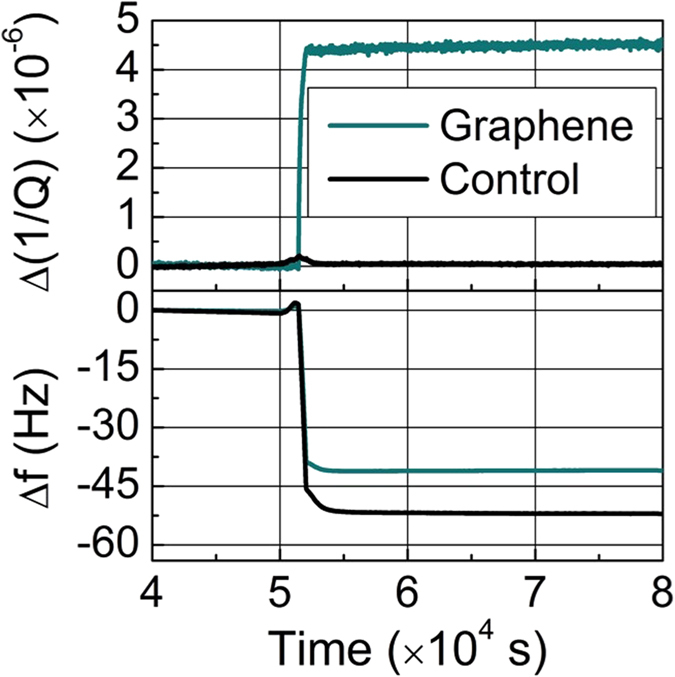
(top) Changes in the dissipation of the control and graphene QCM during gold evaporation. (bottom) The frequency shifts of the control and graphene QCM are plotted as functions of time during gold deposition. The slight fluctuation in the frequency and the dissipation observed prior the opening of the shutter is due to the sensitivity of the QCMs to the radiative heating induced by the evaporator.

Figure 2 shows the influence of electron beam evaporation on the resonance frequencies and energy dissipations of both graphene-coated QCM and the control QCM. The shutter for the evaporator is opened at *t* ≅ 5.2 × 10^4^ sec and gold is deposited for 605 seconds at a rate of 1.6 ng/(cm^2^ sec), as determined by the frequency shift of the control QCM using the Sauerbrey equation^34^. A striking difference can be observed in the energy dissipation at the graphene and control QCM. The dissipation in the graphene QCM is found to increase by 4.45 × 10^−6^ upon gold deposition. On the other hand, the dissipation of the control QCM does not change as observed previously^26^, consistent with inertial mass loading in which the adsorbed gold attaches rigidly.

Precise knowledge of the change in frequency and dissipation enables us to determine the slip time of the gold nanocrystals using the formalism developed for viscous energy dissipation induced by adsorbates on QCMs^25^,^26^. The total frequency shift Δ*f* of the graphene QCM is given by the formula


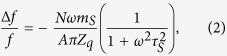


where *f* is the resonant frequency, *N* is the total number of nanocrystals on graphene, *ω* is the angular resonant frequency of the crystal, *m*_*s*_ is the average mass of an individual sliding nanocrystal, *A* is the active area of the crystal, *Z*_*q*_ is the acoustic impedance of AT-cut quartz (8.8 × 10^6^ kg m^−2^ s^−1^), and *τ*_*s*_ = *m*_*s*_/*ξ* is the slip time for sliding crystals, with *ξ* being the drag coefficient. Similarly, the shift in the inverse quality factor of the QCM is


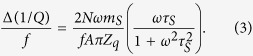


We assume all adsorbed nanocrystals participate in sliding, and, thus, contribute to the observed change in frequency and dissipation, justified by the low defect density of the graphene used for our study. The slip time *τ*_*s*_ can be calculated from the ratio of the shifts in frequency and dissipation for the graphene QCM:


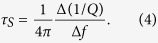


We find the slip time to be 8.9 ns, with an oscillation amplitude of 11.4 nm and a peak surface speed of 35.6 cm/sec using our data. The measured slip time is over an order of magnitude higher than the slip time predicted for a smaller gold nanocrystal with a mass of 1.5 × 10^−22^ kg undergoing the ballistic friction behavior^18^. Therefore, the measured value confirms the enhanced lubricity predicted for the nanocrystals at high surface speeds. The average mass of the nanocrystals can also be determined using the above formula to be 7.7 × 10^−22^ kg. We find that this value is 17% smaller than the value calculated from the amount of gold deposited on the control QCM. Such discrepancy is not surprising since the sticking coefficient of gold on graphite has been determined to be less than 1 below monolayer coverage at room temperature^35^. The average crystal diameter is determined to be 4.2 nm assuming each nanocrystal is spherical with a mass density of 19.3 g/cm^3^, consistent with our AFM measurements.

**Figure 3:**
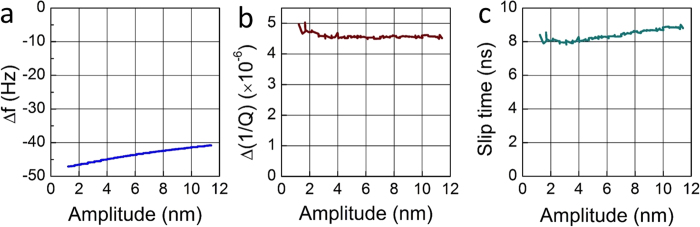
(**a**) Frequency shift, (**b**) change in dissipation, and (**c**) slip time as a function of the oscillation amplitude of the graphene QCM.

Figure 3 shows the dependence of the response of the graphene QCM as a function of the oscillation amplitude, *A*_*osc*_, in the range of 1.5 to 11.4 nm, which is accessible to our QCMs. The peak surface speeds vary from 4.7 to 35.6 cm/sec in this experimentally observable window. The slip time as a function of the oscillation amplitude can be determined again by using *Δf* and *Δ*(*1/Q*) as discussed above. The slip time decreases at lower speeds but the predicted transition to a much lower slip time is not seen. This indicates that the predicted transition to high lubricity occurs below 4.7 cm/sec.

It is important to note that nanocrystals do not collide with each other during our measurements. The motion between gold nanocrystals and graphene QCM can be described by a differential equation^36^:


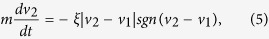


where *v*_*2*_ is the velocity of nanocrystals and *v*_*1*_ is the velocity of graphene QCM. As such, *v*_*1*_ = *v*_*0*_ cos(ωt), where v_0_ is the amplitude of the QCM surface velocity. The solution to this differential equation is given by:


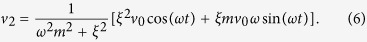


Relative velocity of gold nanocrystal on the graphene QCM is


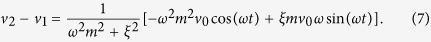


Relative displacement, d, is


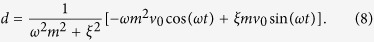


This can be written as


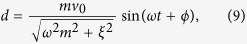


where the phase, ϕ, is given by


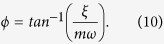


Therefore, the amplitude of the motion of nanocrystals on the graphene QCM is proportional to the maximum amplitude of the surface velocity of the graphene QCM. The maximum sliding amplitude of nanocrystals with respect to graphene is 3.1 nm with the surface velocity amplitude of 35.6 cm/sec, the maximum used for our experiment. The range of diameters of nanocrystals determines the maximum phase angle range. We estimate the range of the phase to be 20 degrees using the diameter distribution of ±1 nm observed in previous measurements with similar deposition parameters^21^. Since the mean distance between nanocrystals in our experiment is 10 nm, the oscillation amplitudes and phase differences between nanocrystals are insufficient to allow collisions.

**Figure 4:**
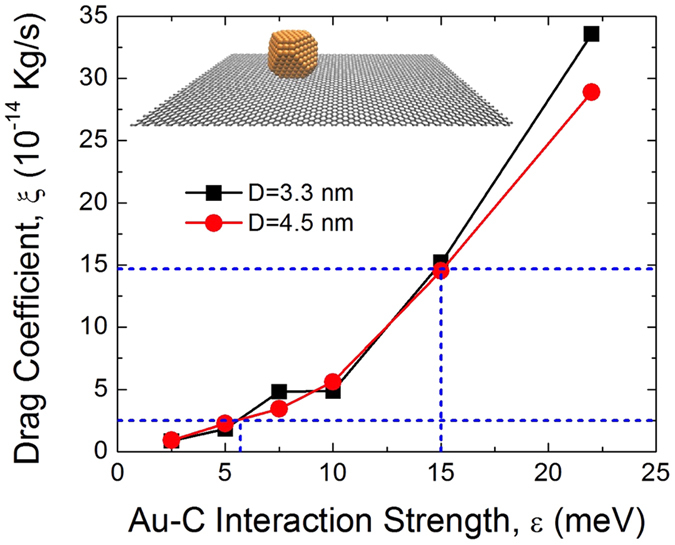
Calculated drag coefficient as a function of the interaction strength between graphene and gold nanocrystals with 3.3 and 4.5 nm diameters; inset is a snapshot of the simulation. Dashed lines indicate the range of the drag coefficients determined from the measured slip time.

The question remains on why we observe the enhanced lubricity that exceeds the previous prediction^18^,^19^. We use molecular dynamics (MD) simulations to determine the answer. Our MD calculations output drag coefficients, which are inversely proportional to slip times. First, our calculations find that the drag coefficient to be independent of size for model nanocrystals ranging in size from 2 to 4.5 nm in diameter; results for two nanocrystals are shown in Fig. 4. Thus, the observed lubricity is not higher than expected because of the larger sizes of our nanocrystals. Instead, our theoretical simulations reveal that the observed lubricity exceeds the prediction due to the interaction strength between graphene and nanocrystals being lower than assumed in previous calculations^18^,^19^. The interaction potential between these surfaces is typically described by the Lennard-Jones potential,


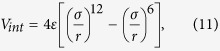


where ε is the depth of the potential well or the interaction strength, σ is the distance at which the interatomic potential is zero, and r is the distance between atoms. The ballistic friction of gold nanocrystals on graphite was predicted using σ = 2.74 Å and ε = 22 meV^18^,^19^. σ and ε are expected to be dependent on materials and have not been determined experimentally for either the gold-graphene or gold-graphite interfaces. Previous theoretical calculations for the gold-graphite interface have used ε ranging from 12.7 to 34.1 meV and σ ranging from 2.74 to 3.00 Å^37^,^38^,^39^. We focus on ε and how it affects the drag coefficient rather than on σ because a larger range of ε is deemed reasonable for the gold-graphite system and σ, with a narrower acceptable range, was found to have a much less significant effect in our preliminary MD simulations. As shown in Fig. 4, the drag coefficient *ξ* increases with the interaction strength and ε being lower than 22 meV can explain the observed lubricity that exceed the previous prediction. Since the measured quantities determine the slip time rather than the drag coefficient, we calculate the drag coefficient using the average mass and slip time as well as the expected variation in diameters. We find the drag coefficient to be 8.6 ± 6.1 × 10^−14^ kg/s at the peak surface speed of 35.6 cm/sec. Therefore, our simulation reproduces the experimentally-measured drag with ε ≈ 6 ~ 15 meV as shown in Fig. 4. We note that the interaction strengths are condition and material dependent^30^,^40^ and the values obtained here are not generally-applicable for graphene on arbitrary substrates. We conclude that the weaker interaction strength than previously anticipated in the prediction of the ballistic nanofriction is the cause for the observed high lubricity of nanocrystals.

In summary, we have measured the changes in frequency and dissipation induced by depositing gold nanocrystals on QCMs covered with graphene. Nanocrystals increase the dissipation of the QCM coated with graphene while leaving the dissipation of the control QCM without graphene unaffected. The measured change in dissipation and frequency induced by the gold nanocrystals can be understood in the framework developed for friction of adatoms on metal surfaces. The determined slip time of gold nanocrystals is found to be over an order of magnitude higher than the value predicted for the ballistic friction of gold nanocrystals on graphite, confirming the enhanced lubricity predicted at high surface speeds. We perform molecular dynamics calculations and show that the observed lubricity, which exceeds the previous prediction, is due to the interaction strength between gold nanocrystals and graphene being lower than estimated in the previous modeling studies. As discussed above, specific sliding distances can be inferred from our analysis of the experimental data. *In-situ* high-resolution microscopy should be able to directly confirm these distances and verify our analysis. Furthermore, the precision of our measurement of the drag coefficient is limited by the distribution in the size of nanocrystals. Future experiments with more control over nanocrystals will improve the capability to determine their drag coefficients using the QCM technique.

## Methods

The control and graphene QCM are as 5 MHz AT-cut QCM blanks (ICM Mfg.). Electrodes are patterned on both sides using a 10 nm Cr adhesion layer followed by 100 nm of Cu, and then 80 nm of Au using thermal evaporation. The graphene-coated QCM is prepared by transferring graphene grown by low-pressure chemical vapor deposition on copper^41^ onto a metallized QCM using a conventional PMMA-assisted, wet transfer method. As such, graphene is on unreconstructed, polycrystalline gold surface. Both QCMs are then annealed in H_2_/Ar at 350 °C for 3 hours at atmospheric pressure in order to clean the surfaces of the QCMs^42^. Raman measurements produce an I_dg_ ratio of 0.074 and a graphene defect density of 1.9 × 10^10^ defects/cm^2^ 43. The QCMs are finally placed into a UHV chamber with a base pressure of ~2 × 10^−9^ Torr. The crystals are vacuum annealed *in situ* at 180 °C for 8 hours prior to experiments.

QCM experiments are performed in UHV. Nanocrystals are formed by evaporating gold on graphene via e-beam evaporation^44^. The control and graphene QCMs are placed next to each other and receive the same flux of gold. Both QCMs are driven at their fundamental frequency *f*_*F*_ ≈ 5 *MHz*. A previous study found that graphene nanoribbons, which are ~100 nm^2^ in size, exhibit high lubricity on reconstructed, well-ordered gold surfaces and that the reconstruction was critical to the observed effect^7^. We assume that graphene does not slide on QCMs because of their unreconstructed, polycrystalline and disordered gold surfaces.

Frequency and electronic amplitude are continuously monitored for both QCMs using an Agilent 53220A frequency counter. Quality factors of both the control and graphene QCM are determined by ring-down measurements using a Tektronix 2024B oscilloscope. The ring down time constant *τ* is extracted from the ring down curve and *Q* is determined via the relationship *Q* = *πτf*, where *f* is the fundamental frequency of the QCM.

To image gold nanocrystals on graphene with TEM, gold is deposited onto CVD graphene on electron-transparent substrates in the same manner as described above for the QCM devices. The substrates consist of Si with thermally grown SiO_2_ (800 nm) and Si_3_N_4_ (16 nm) in which thin (<1 um) windows are etched with KOH. After deposition of the graphene and gold, the bottom side of the windows is etched by placing the chip over HF vapor to remove the oxide, leaving a thin nitride membrane. Samples are imaged in a Titan 80–300 FEG S/TEM at 300 kV at high spot size (low beam current) to minimize beam-induced damage on the sample.

AFM images are acquired in air at room temperature using a Bruker Dimension 5000 atomic force microscope operating in tapping mode in ambient conditions. We use the Supersharp silicon AFM tips fabricated by Mikromasch with tip radius of <1 nm for imaging (Mikromasch Hr’Res-10/Cr-Au).

The all atom simulations consist of a single gold nanocrystal on graphene. The nanocrystal is modeled as Au atoms in a truncated-octahedron geometry with approximate diameters between 2 and 4.5 nm. The nanocrystal is placed such that its (111) surface is in contact with graphene that has in-plane dimensions of 14.0 nm × 7.1 nm. The interatomic interactions within the gold are described by the Embedded Atom Model (EAM)^45^ and those within the graphene described by the Adaptive Intermolecular Reactive Empirical Bond Order (AIREBO) potential^46^. The Lennard-Jones potential is used to model gold-graphene interactions and is given by Equation 11, V_int_ = 4ε[(σ/r)^12^ − (σ/r)^6^], where ε is the interaction strength, σ is the distance at which the interatomic potential is zero, and *r* is the distance between atoms. We ran simulations at a range of interaction strengths, ε, to explore the effect of this parameter. The simulations are performed using LAMMPS simulation software^47^ with a time step of 1 fs, and the Nosé-Hoover thermostat is used to control the temperature at 300 K.

First, simulations are run with no externally applied force to characterize the diffusion behavior of the system. Diffusion speed for each nanoparticle diameter is calculated from the approximately linear slope of the increase in the center of mass displacement of the nanocrystal with time. During production simulations, the graphene is translated laterally at speeds between 10 and 500 m/s. The center of mass of the gold nanocrystal and force acting on it are recorded and then averaged over time intervals of 0.5 ns. The drag coefficient is then calculated from ξ = F/∆v, where F is the average force on the gold nanocrystal in the direction that the graphene is translated and Δv is the difference between the sliding speed of the nanoparticle and the graphene in that same direction. The drag coefficient is calculated from the simulation only when the difference in the speed of the nanocrystal and graphene is greater than the diffusion speed for a given nanoparticle size, i.e. when the nanocrystal is sliding relative to the graphene.

## Additional Information

**How to cite this article**: Lodge, M. S. *et al*. Lubricity of gold nanocrystals on graphene measured using quartz crystal microbalance. *Sci. Rep.* **6**, 31837; doi: 10.1038/srep31837 (2016).
